# Case Report: Rare presentation of adult intussusception at Orotta National Referral Hospital, Eritrea

**DOI:** 10.12688/f1000research.17670.2

**Published:** 2021-04-21

**Authors:** Senay Iyassu, Million Abraha

**Affiliations:** 1 Department of Surgery, Orotta National Referral Hospital, Asmara, Eritrea; 2 Faculty of Medicine, Institute of Social and Preventive Medicine (ISPM), Bern, 3012, Switzerland; 3 Fairmed, Health for the Poorest, Bern, 3001, Switzerland

**Keywords:** Intussusception, Bowel obstruction, Abdominal pain

## Abstract

A 38-year-old woman presented at Orotta National Referral Hospital emergency department in May 2017 with pain in the epigastric region and vomiting. Physical examination revealed no pertinent findings. Blood and urine tests were normal, and erect abdominal x-ray revealed a distended small intestine with multiple layers of “air-fluid levels”. CT scan and MRI were not done due to their temporary unavailability. During laparotomy a large mass of 20x20 cm in size was detected in the mid-jejunum of the small intestine. This leading tumor caused intussusception and coiling of the small intestine. As there are no typical symptoms of intussusception, it is very important to do CT scan for patients with long-standing abdominal pain and vomiting to achieve a definitive diagnosis of intussusception.

## Introduction

Intussusception is commonly defined as telescoping of a proximal part of an intestinal loop towards the distal part of the loop ^1– 6^. The incidence of intussusception is 1–3 cases per 1,000,000 population per year ^2, 7^. The condition is more frequent in children than in adults ^1^. Patients present sometimes with nonspecific symptoms ^8, 9^. The majority of patients come to hospital with abdominal pain and vomiting ^9^. As these are unspecific symptoms, diagnosis of intussusception could be missed or even delayed before one would entertain intussusception as a cause of abdominal pain ^10^. The unspecific presentation makes it difficult to define clearly the diagnosis before surgical operations ^1, 11^. Ordinarily, half of these cases are diagnosed in theater ^12^. Helpful diagnostic tests include plain abdominal film ^13, 14^, abdominal CT scan ^15^ and ultrasound ^16^. Operative procedures to reduce the intestine is the treatment modality of intussusception ^1, 17^. In this paper we present a patient with intussusception with nonspecific symptoms who presented to Orotta National Referral Hospital, and emphasize the importance of early CT scan for quick and definitive diagnosis.

## Case report

### Presentation

A 38-year-old female patient presented to Orotta National Referral Hospital, Asmara, Eritrea, in May 2017 with abdominal pain and vomiting of 3-month duration. The pain was diffuse, intermittent and more on the periumbilical area. She only vomited after ingesting food, otherwise she does not. There is no history of abdominal distension and she was passing gas. Her condition gradually worsened from time to time. There was no history of fever, no bloody vomiting, and no history of jaundice or urinary complaints. There was no known family history of intestinal obstruction.

On physical examination, the patient appeared sick and emaciated, although vital signs where within normal limits. She had slightly pale conjunctiva and dry mucosa. She had good air entry on both lungs with normal heart sound. There was diffuse tenderness on the abdomen but more on the epigastric region. There was no guarding or rebound tenderness. There was no any palpable mass on the abdomen until 1 day before the operation, when a large suprapubic mass was detected. Bowel sounds were present.

Laboratory findings showed that complete blood count was within the normal limit. Hemoglobin of 10 g/dl (normal range, 12–16 g/dl), white blood cell count of 7×10 ^3^/µl (normal range, 5–10 × 10 ^3^/μl), platelet count of 350×10 ^3^/µl (normal range, 150–400 × 10 ^3^/μl). Urinalysis reveled no abnormal finding and blood chemistry values were aspartate transaminase levels of 57 U/l serum (normal range, 8–48 U/L), alanine aminotransferase level of 43 U/l (normal range, 7–55 U/l), amylase of 191 U/l (normal range, 23–85 U/l). Several plain abdominal x-rays were done with no pertinent findings. The last time plain abdominal x-ray was done showed a distended small intestine and with multiple “air-fluid levels”. Ultrasound of the abdomen depicted pockets of fluid collections. No abnormality was observed upon gastroscopic study (mild duodenal ulcer with excessive bile reflux). CT scan and MRI were not available.

Despite the diagnostic challenges, several differential diagnoses were entertained, such as chronic peptic ulcer disease cholecystitis acute pancreatitis and partial intestinal obstruction and was managed conservatively. The patient was being treated as case of peptic ulcer disease before her surgical operation and was taking amoxicillin 1g twice daily and metronidazole 500mg twice daily for 10 days.

### Surgical intervention

**Figure 1.**
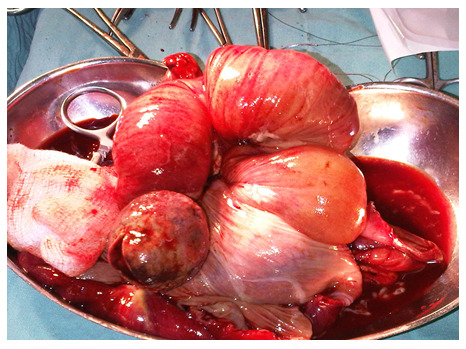
Resected bowel.

**Figure 2.**
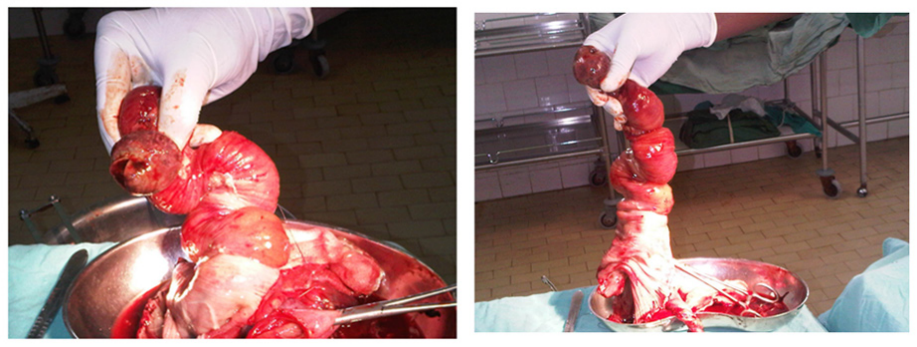
Mid jejunum with a leading tumor.

At 1 day before surgery, an abdominal mass was felt during repeated physical examination. Non-operative reduction using hydrostatic or pneumatic pressure by enema was not attempted. Surgical intervention was decided and performed 2 weeks after initial presentation, with an impression of small intestine obstruction secondary to malignancy. The patient was kept nothing per mouth (NPO) and was given normal saline intravenous fluid, broad-spectrum ceftriaxone (1 g intravenous twice a day) and metronidazole (500 mg intravenous three times a day). She was also given hydrocortisone (100 mg intravenous three times a day for 3 days). The patient was also administered 2 units of blood were before surgery. During laparotomy a large mass of 20×20 cm was identified in mid jejunum and a leading tumor had caused intussusception and coiling of the small intestine ( Figure 1 and Figure 2). During laparotomy, intussusception reduction was attempted manually but the large intestine was too greatly looped, tangled and edematous to be reduced so resection was decided. Around 1 meter of the bowel was resected, and anastomosis was done in two layers. A biopsy of the leading tumor later showed it to be a simple hemangioma of the small intestine. In her postoperative management, she was kept NPO for 3 days and took intravenous fluids. Ceftriaxone and metronidazole were continued for 10 days. On her second day postoperatively, abdominal auscultation revealed the start of bowel sounds. She was discharged 2 weeks after surgery and was appointed to come for her follow-up after a month. In her follow up visit full remission was confirmed.

## Discussion

The preoperative diagnosis of intussusception remains challenging as the patients usually have nonspecific symptoms during their presentation to medical facilities ^11^. CT scans aid diagnosis considerably and usually shows a “bull’s-eye” sign ^18, 19^. Early in her presentation, the patient did not have signs of intestinal obstruction, but because of her persistent vomiting and epigastric pain other diagnoses were suggested. The diagnosis was greatly delayed partly because of diagnostic constraints. The temporary unavailability of the only CT scanner in the whole country made it difficult for early and accurate diagnosis. Looking at the importance of CT scan and MRI in the diagnosis of many medical and surgical conditions, it is highly recommended that the Eritrean Ministry of Health should import several additional CT scans or MRIs for a successful diagnosis and management of such health conditions. It is recommended as well to use CT scan as early as possible when a patient is suffering from a long-standing abdominal pain and vomiting.

## Data availability

No data is associated with this article.

## Consent

Written informed consent for publication of their clinical details and clinical images was obtained from the patient.
