# Supplementary material for: Case Report: Rare presentation of adult intussusception at Orotta National Referral Hospital, Eritrea
Source: F1000Res. 2021 Apr 21;8:55. Originally published 2019 Jan 14. [Version 2] doi: 10.12688/f1000research.17670.2 (PMC8080982; doi:10.12688/f1000research.17670.2)
Supplement: Peer review history

# Peer review: Reviewer response for version 2

**DOI:** 10.5256/f1000research.56271.r83681

**Published:** 2021-04-22

**Authors:** Yifan Wang

**Affiliations:** 1 The Research Institute, McGill University Health Centre, Montreal, QC, Canada

I now approve this article for indexing.

Are enough details provided of any physical examination and diagnostic tests, treatment given and outcomes?

Partly

Is the case presented with sufficient detail to be useful for other practitioners?

Yes

Is sufficient discussion included of the importance of the findings and their relevance to future understanding of disease processes, diagnosis or treatment?

Partly

Is the background of the case’s history and progression described in sufficient detail?

Yes

Reviewer Expertise:

Gastrointestinal surgery

I confirm that I have read this submission and believe that I have an appropriate level of expertise to confirm that it is of an acceptable scientific standard.

# Peer review: Reviewer response for version 1

**DOI:** 10.5256/f1000research.19322.r82133

**Published:** 2021-04-13

**Authors:** Gennaro Martines

**Affiliations:** 1 Department of Emergency and Organ Transplantation, University "Aldo Moro" of Bari, Bari, Italy

Intussusception is 1-5% of causes of intestinal ileus and double intussusception is an even more exceptional observation. CT and MRI are the gold standard for diagnosis but ultrasonography can be an effective diagnostic tool and demonstrates multiple concentric rings associated with an abdominal echogenic mass and echolucent rings representing the mucous covering of the intussuscepted bowel segment.

 I think that this description is due in the presentation of ultrasonography. for the differential diagnosis.

 Some English errors like bowl in figure 1.

 Add in references this article which well describe a case like this:

 Fabio Marino ^1 ^, Pierluigi Lobascio, Gennaro Martines, Angela D Di Franco, Marcella Rinaldi, Donato F Altomare Double jejunal intussusception in an adult with chronic subileus due to a giant lipoma: a case report Chir Ital Mar-Apr 2005;57(2):239-42. ^1^

Are enough details provided of any physical examination and diagnostic tests, treatment given and outcomes?

Yes

Is the case presented with sufficient detail to be useful for other practitioners?

Yes

Is sufficient discussion included of the importance of the findings and their relevance to future understanding of disease processes, diagnosis or treatment?

Yes

Is the background of the case’s history and progression described in sufficient detail?

Partly

Reviewer Expertise:

General surgery

I confirm that I have read this submission and believe that I have an appropriate level of expertise to confirm that it is of an acceptable scientific standard.

## References

[1] : Double jejunal intussusception in an adult with chronic subileus due to a giant lipoma: a case report. *Chir Ital*.57(2) :239-42 15916153

# Peer review: Reviewer response for version 1

**DOI:** 10.5256/f1000research.19322.r58473

**Published:** 2020-02-17

**Authors:** Yifan Wang

**Affiliations:** 1 The Research Institute, McGill University Health Centre, Montreal, QC, Canada

Lyassu *et al.* describe a rare case of adult small bowel-small bowel intussusception caused by an intestinal hemangioma that was treated with surgical resection.

 The diagnosis was challenging because of the lack of institutional availability of cross-sectional imaging (CT/MRI). It would be informative to add an image of the abdominal x-ray showing air-fluid levels. Also, it is unclear what the "pockets of fluid collections" visualized on abdominal ultrasound represent.

 It would be interesting to show histopathological correlation of the intestinal hemangioma (H&E section). There are only a handful of such cases causing intussusception in the literature, and the authors may want to summarize this experience in the discussion.

 Figure 1. legend should read "bowel", rather than "bowl".

Are enough details provided of any physical examination and diagnostic tests, treatment given and outcomes?

Partly

Is the case presented with sufficient detail to be useful for other practitioners?

Yes

Is sufficient discussion included of the importance of the findings and their relevance to future understanding of disease processes, diagnosis or treatment?

Partly

Is the background of the case’s history and progression described in sufficient detail?

Yes

Reviewer Expertise:

Gastrointestinal surgery

I confirm that I have read this submission and believe that I have an appropriate level of expertise to confirm that it is of an acceptable scientific standard, however I have significant reservations, as outlined above.
